# Whole-genome analysis of the recombination and evolution of newly identified NADC30-like porcine reproductive and respiratory syndrome virus strains circulated in Gansu province of China in 2023

**DOI:** 10.3389/fvets.2024.1372032

**Published:** 2024-04-12

**Authors:** Shoude Jiao, Jing Zhang, Jian Wang, Xueqing Ma, Guoxiu Li, Jiaoyang Li, Zhanding Cui, Dong Li, Pinghua Li, Qiaoying Zeng, Zaixin Liu, Zengjun Lu, Pu Sun

**Affiliations:** ^1^State Key Laboratory for Animal Disease Control and Prevention, College of Veterinary Medicine, Lanzhou University, Lanzhou Veterinary Research Institute, Chinese Academy of Agricultural Sciences, Lanzhou, China; ^2^Gansu Province Research Center for Basic Disciplines of Pathogen Biology, Lanzhou, China; ^3^Department of Preventive Veterinary Medicine, College of Veterinary Medicine, Gansu Agricultural University, Lanzhou, China

**Keywords:** porcine reproductive and respiratory syndrome virus, ORF5, NSP2, NADC30-like, recombination

## Abstract

Porcine reproductive and respiratory syndrome virus (PRRSV) remains one of the major threats to swine industry, resulting in huge economic losses worldwide. Currently, PRRSV has diversified into multiple lineages with characteristics of extensive recombination in China. In this research, three virus strains were isolated and four virus whole genome sequences were generated and analyzed from clinical samples collected in Gansu province of China in 2023. The four virus strains were designated GSTS4-2023, GSLX2-2023, GSFEI2-2023 and GSBY4-2023. Phylogenetic analysis based on ORF5 sequences showed that GSTS4-2023, GSLX2-2023, GSFEI2-2023 and GSBY4-2023 shared 91.7, 91.2, 93.2 and 92.9% homology with NADC30 strain respectively, and belonged to lineage 1 of PRRSV-2. In addition, one amino acid deletion was observed at position 33 in ORF5 of GSTS4-2023, GSLX2-2023 and GSFEI2-2023. Moreover, amino acid alignment of the four strains showed a typical discontinuous 131-amino acid (aa) deletion in NSP2 for NADC30-like virus strains. Recombination analysis revealed that all four strains originated from NADC30 (lineage 1), with their minor parents coming from JXA1-like strains (lineage 8), VR-2332-like strains (lineage5) and QYYZ-like strains (lineage3). Finally, the three isolated virus strains, GSTS4-2023, GSLX2-2023 and GSFEI2-2023 showed relatively low levels of replication in cell culture. Our findings provide important implications for the field epidemiology of PRRSV.

## Introduction

Porcine reproductive and respiratory syndrome (PRRS) is characterized by respiratory and reproductive disorders in swine, causing huge economic losses in the past several decades. The etiologic agent, porcine reproductive and respiratory syndrome virus (PRRSV), was first reported in North Carolina and considered to be one of the primary pathogens affecting swine industry (1). Immunosuppression is an inevitable impact of PRRSV infection because the destruction of porcine alveolar macrophages (PAM) leads to the occurrence of secondary infections (2). More seriously, recombination and mutation, the two common phenomena in RNA viruses, are undesirable events that constantly alter the genome sequence and may increase PRRSV virulence (3–5). Therefore, PRRSV needs to be continuously monitored in different areas to contribute to local PRRS prevention and control.

The PRRSV genome is about 15 kb in length and comprises a single-stranded, positive-sense RNA molecule that encodes at least 16 non-structural and 8 structural proteins (6). Due to its high degree of genetic diversity, PRRSV has been further divided into two species, PRRSV-1 (formerly known as European genotype 1) and PRRSV-2 (formerly known as North American genotype 2) (7). PRRSV-1 and PRRSV-2 strains originated from Lelystad virus and VR-2332 respectively, and they shared relatively low genetic homology with each other (only 50–70%) (6, 8, 9). In China, PRRSV-2 is currently the most prevalent genotype on farms and is further divided into lineage 1 (represented by NADC30 and NADC34), lineage 3 (represented by QYYZ), lineage 5 (represented by VR-2332), and lineage 8 (represented by JXA1) according to the widely accepted PRRSV classification system and ORF5 sequence (10–13). CH-1a was first isolated in 1996 in China, and it was the crucial original strain that generated various mutants (14). Since then, various PRRSV strains have been isolated and identified. Before 2006, mild-to-moderate reproductive disorder was the main clinical manifestations of PRRSV in China, however severe fever with a high mortality rate emerged and spread widely to a number of provinces and municipalities thereafter (15–17). A number of novel strains of PRRSV-2 have been characterized and named “HP-PRRSV” due to theirs high pathogenicity (15). It has been reported that these strains originated from CH-1a with a 30 amino-acid discontinuous deletion in the NSP2 gene by whole-genome analysis (15, 18, 19). QYYZ-like strain was first isolated in 2010 and mostly confined to southern China (20, 21). Thereafter, NADC30-like strains and NADC34-like strains, which had higher propensity for recombination with a discontinuous 131-amino-acid deletion and 100-amino-acid deletion in the NSP2 gene respectively, had become the prevalent strains in China (12, 16, 22, 23). Recombination among strains from different lineages is becoming a common event, especially in NADC34-like, NADC30-like, QYYZ-like and HP-PRRSV-like strains (24–27).

The prevention and control of PRRS is becoming increasingly difficult, as novel PRRSV variants keep emerging. The new strains, especially NADC30-like strains, may cause severe symptoms and continuously challenge the pig industry. Here, we report the information on genomic recombination and evolution of four field virus strains detected in 2023, which will enrich our knowledge on PRRSV epidemiology and contribute to the prevention and control of PRRSVs.

## Materials and methods

### Sample collection, PRRSV detection and whole-genome sequencing

Samples (including serum, lymph node, and lung samples) from clinically suspected PRRS onset pigs were collected from different swine farms in Tianshui, Linxia, Zhangye and Baiyin respectively in Gansu province in China, and further grown within RPMI 1640 medium under low temperature to make tissue homogenate. Supernatant was collected after centrifuge at 3,000 × g for 20 min at 4°C for subsequent experiments. Total RNA was extracted using a commercial RNA extract kit (OMEGA, USA) for subsequent tests. For the PRRSV detection, specific primers for the amplification of the viral ORF5 gene were designed. In addition, eight pairs of primers were designed to amplify the whole genome of PRRSVs. RT-PCR was carried out according to the instruction of Vazyme HiScript II One Step RT-PCR Kit (Vazyme, China). To obtain the sequence of the untranslated region (5′ UTR and 3′ UTR), four pairs of primers were synthesized to perform 5′ RACE and 3′ RACE using Vazyme HiScript-TS 5′/3’ RACE Kit (Vazyme, China). All of the primers were synthesized by Qingke Biotechnology (Qingke, China) (see Supplementary Tables S1–S3). Sequencing was conducted using the Sanger sequencing approach. Whole genome sequences were *de novo* assembled by the Lasergene software package (DNASTAR, USA). In addition, N-glycosylation sites of GP5 protein were predicted using NetNGlyc.^1^

### Phylogenetic analysis

Thirty-eight reference strains were downloaded from NCBI for the molecular evolutionary analysis (see Supplementary Table S4). The phylogenetic trees were constructed with neighbor-joining method of MEGA11 software (MEGA11, USA) based on the ORF5, NSP2, ORF3 and whole-genome sequences. The reliability of our data was evaluated using bootstrapping with 1,000 replicates. All the strains were additionally annotated with associated lineages. In addition, ChiPlot was used to annotate the phylogenetic trees.^2^

### Recombination analysis

VR-2332, JXA1, QYYZ, NADC30, and IA/2014/NADC34 were selected as PRRSV reference strains. Recombination events were initially detected by Recombination Detection Program version 4.67 (RDP4). Potential recombination was identified and further verified by SIMPLOT (version 3.5.1, USA). The four new PRRSV sequences were selected as the query sequence. All the recombination analyses were executed with default settings.

### Virus isolation, TCID_50_ determination and quantitative RT-PCR (RT-qPCR)

PAM cells were prepared and stored as previously described (28). The PRRSV-positive specimens were inoculated onto PAMs for virus isolation. Cells were cultured with RPMI 1640 medium (GIBCO) and maintained at 37°C. Daily cytopathic effects (CPE) observation was performed to permit virus invasion at the first three passages. The PRRSVs were then inoculated onto Marc-145 cells for infection, and virus-infected Marc-145 cells were screened with indirect immunofluorescence assay (IFA) at the third passage. In brief, the infected Marc-145 cells were fixed in 4% paraformaldehyde buffer after washing with PBS, and then treated with 3% bull serum albumin (BSA, Sigma, USA) solution to seal off the nonspecific binding site. IFA employed diluted antibody against nucleocapsid protein (Npro) as the primary antibody and FITC coupled Monoclonal mouse Anti-Swine IgG as the second antibody. In addition, DAPI was used to achieve nucleus visualization. The fluorescence imaging was performed on a fluorescence imaging system. PRRSV genomic fragments were amplified at the fourth passage and harvested by three freeze–thaw cycles. In order to accomplish TCID_50_ determination, Marc-145 cells were plated in 96-well plates and cultured in High-glucose DMEM (GIBCO). Tenfold serially diluted virus supernatants were added to each well in 8 replicates. TCID_50_ was calculated with the Reed-Muench formula (29). For RT-qPCR, total RNA was isolated from PRRSV-infected Marc-145 cells. The amplification condition was 42°C for 5 min, 95°C for 10 s, followed by 40 cycles of 95°C for 5 s and 60°C for 34 s. Primers and TaqMan probe used for RT-qPCR are available in Supplementary Table S5.

## Results

### The new strains belong to PRRSV-2 and exhibit different genomic characterization

The complete sequences of GSTS4-2023, GSLX2-2023, GSFEI2-2023 and GSBY4-2023 were 15,061 nt, 15,025 nt, 15,014 nt and 14,981 nt in length respectively, including 5′ untranslated region and 3′ untranslated region. Whole genome homology of GSTS4-2023, GSLX2-2023, GSFEI2-2023, and GSBY4-2023 to reference strains VR-2332 (lineage5), JXA1 (lineage8), QYYZ (lineage3), NADC30 (lineage1.8) and IA/2014/NADC34 (lineage1.5) were greater than 80% (Table 1). Such a high degree of sequence homology indicates that all of the four strains belong to PRRSV-2.

**Table 1:** Nucleotide identities of GSTS4-2023, GSLX2-2023, GSFEI2-2023 and GSBY4-2023 compared with the reference strains.

Region	Nucleotide identity % (GSTS4-2023/GSLX2-2023/GSFEI2-2023/GSBY4-2023)				
	VR-2332	JXA1	QYYZ	NADC30	IA/2014/NADC34
Full-length	85/86/84/85	86/84/83/86	82/82/81/84	89/91/92/89	84/84/84/84
5′ UTR	91/93/89/90	100/94/90/96	95/92/91/92	93/94/96/89	90/93/92/87
ORF1a	83/82/81/83	85/81/79/84	80/78/77/80	86/90/90/86	80/80/80/80
Nsp1	87/84/82/86	93/83/81/93	86/80/80/87	83/92/91/83	82/83/83/82
Nsp2	78/78/78/78	76/75/76/75	74/73/73/73	89/90/89/89	76/76/77/76
Nsp3	87/83/83/87	89/81/80/86	87/82/80/85	89/91/90/89	87/83/83/86
Nsp4	88/86/84/88	92/85/83/95	91/85/84/94	84/92/91/82	84/82/82/85
Nsp5	85/86/84/84	92/85/83/95	91/85/84/94	84/92/91/82	84/82/82/85
Nsp6	87/92/85/92	88/92/85/96	85/94/83/94	81/98/92/90	83/88/81/88
Nsp7	86/86/83/87	92/91/79/93	88/87/78/89	79/85/92/81	79/81/81/81
Nsp8	95/91/87/89	96/96/84/92	92/92/82/90	88/87/91/82	91/92/87/87
ORF1b	87/93/87/86	88/93/86/86	85/91/84/85	91/91/94/93	87/90/87/87
Nsp9	88/93/87/87	91/90/86/87	87/87/84/86	89/89/94/91	87/87/88/87
Nsp10	85/85/85/86	84/84/84/85	83/85/84/85	93/93/94/93	89/88/89/88
Nsp11	87/90/88/87	87/92/94/89	85/86/87/86	93/91/91/93	84/85/86/85
Nsp12	85/87/87/86	87/87/85/87	82/85/82/86	91/94/94/93	85/85/84/82
ORF2	88/88/89/89	87/85/86/89	86/85/85/95	92/92/92/85	85/84/85/85
ORF3	83/91/90/86	84/87/86/88	82/84/85/94	95/89/88/83	85/87/86/83
ORF4	87/86/87/87	85/85/85/90	85/85/84/93	94/96/95/87	92/94/94/87
ORF5	84/85/85/85	84/85/85/84	82/84/83/83	92/91/93/93	87/88/88/87
ORF6	87/90/90/88	87/90/90/87	88/90/89/88	95/95/95/96	93/93/92/93
ORF7	89/88/91/90	87/87/89/89	85/86/87/86	95/93/94/95	94/92/93/94
3′ UTR	92/90/92/93	91/87/89/90	90/86/88/89	95/97/96/95	93/94/95/94

The 5’ UTR, Nsp1, NSP4-9 of GSTS4-2023 shared 84.7–94.8% nucleotide homology with JXA1, which were higher than those shared with other selected strains, and its Nsp10-12, ORF2-7 and 3’ UTR shared 91.0–97.9% sequence identity with NADC30. The 5’ UTR, ORF1a, NSP1-6, NSP10, NSP12, ORF2, ORF5-7 and 3’ UTR of GSLX2-2023 strain shared the highest sequence identity with NADC30 (91–95.3%) compared to other reference strains, whereas its NSP1, NSP7, NSP8 and NSP11 shared the highest sequence identity with JXA1 (89.8–97.9%). NSP9 and ORF1b of GSLX2-2023 exhibited 92.7 and 93.3% sequence identity with VR-2332, respectively, which were higher than those shared with other representative strains. The 5’ UTR, ORF1a (NSP1-8), ORF1b (NSP9, NSP10, NSP12), ORF4-7 and 3’ UTR of GSFEI2-2023 shared the highest sequence identity with NADC30 (89.2–95.8%), while NSP11 and ORF3 exhibited the highest sequence identity with JXA1 (93.6%) and VR-2332 (86.3%) respectively. For GSBY4-2023, 5’ UTR, NSP1 and NSP4-8 shared the highest sequence identity with JXA1 (91.9–96.2%), whereas ORF1a (NSP2, NSP3), ORF1b (NSP9-12), ORF5-7 and 3’ UTR exhibited the highest sequence identity with NADC30 (85.9–95.8%). Notably, ORF2-4 of GSBY4-2023 shared the highest sequence identity with QYYZ (92.5–95.2%), indicating the potential existence of QYYZ-like strains in Gansu.

### The new strains are classified as NADC30-like strain based on ORF5, and showed different genomic characteristics based on NSP2, ORF3 and their whole-genomes

**Figure 1:**
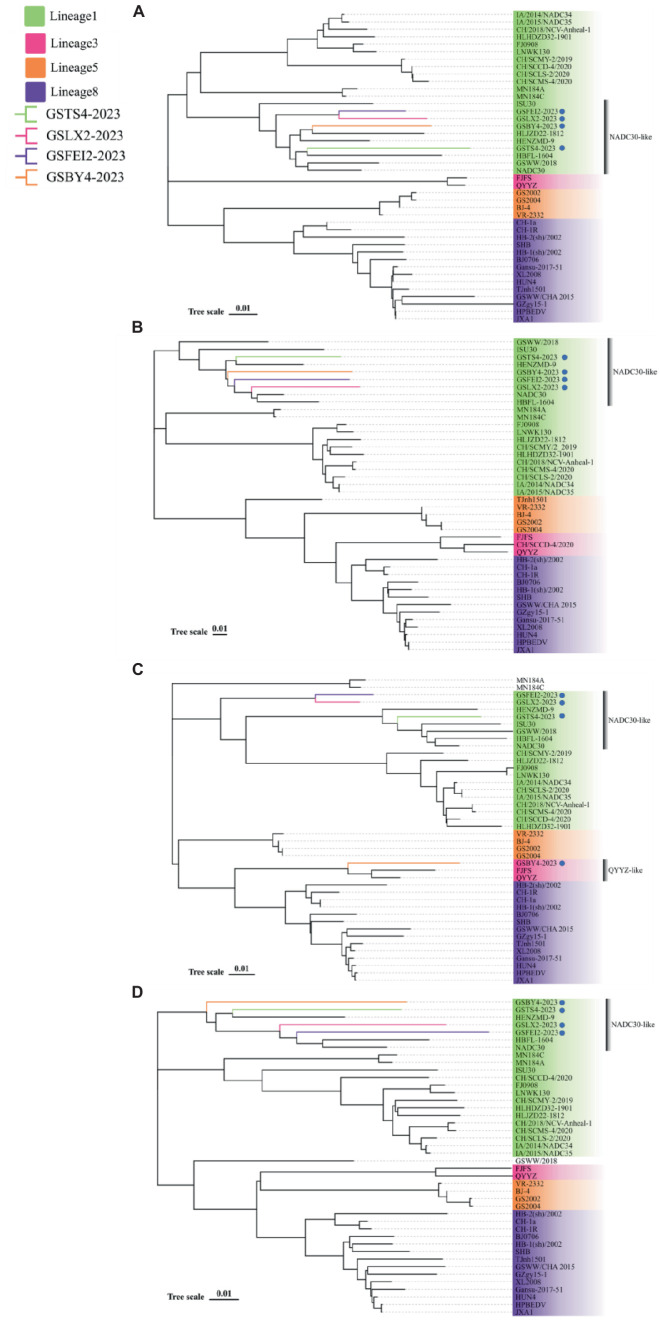
Phylogenetic trees based on ORF5 **(A)**, NSP2 **(B)**, ORF3 **(C)** and full-length genome **(D)** of GSTS4-2023, GSLX2-2023, GSFEI2-2023 and GSBY4-2023 alongside 38 PRRSV reference strains obtained from GenBank. The four strains were annotated with “
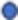
” Strains from lineage 1, lineage 3, lineage 5 and lineage 8 were marked with block in green, pink, orange and purple, respectively.

To determine the genetic relationship between the four strains and other PRRSV isolates, we constructed four phylogenetic trees based on their ORF5, NSP2, ORF3 and whole-genome sequences, respectively. Phylogenetic tree based on the ORF5 gene showed that GSTS4-2023, GSLX2-2023, GSFEI2-2023 and GSBY4-2023 are NADC30-like strains belonging to lineage 1. Simultaneously, GSLX2-2023 and GSFEI2-2023 were located on the same minor branch in lineage1, based on ORF5 gene (Figure 1A). In addition, the phylogenetic tree of NSP2 classified the four strains into four different minor branches that belonged to NADC30-like strains (Figure 1B). As for phylogenetic tree based on ORF3 genotyping, GSTS4-2023, GSLX2-2023 and GSFEI2-2023 belonged to NADC30-like strains, and GSLX2-2023 and GSFEI2-2023 were classified into the same minor branch belonging to lineage 1 (Figure 1C). Interestingly, GSBY4-2023 had the closest relationship with QYYZ, which was mainly located in southern China (Figure 1D). The four strains were NADC30-like strains belonging to lineage 1 based on their whole genome tree.

### Amino acid deletions identified in ORF5 and NSP2

**Figure 2:**
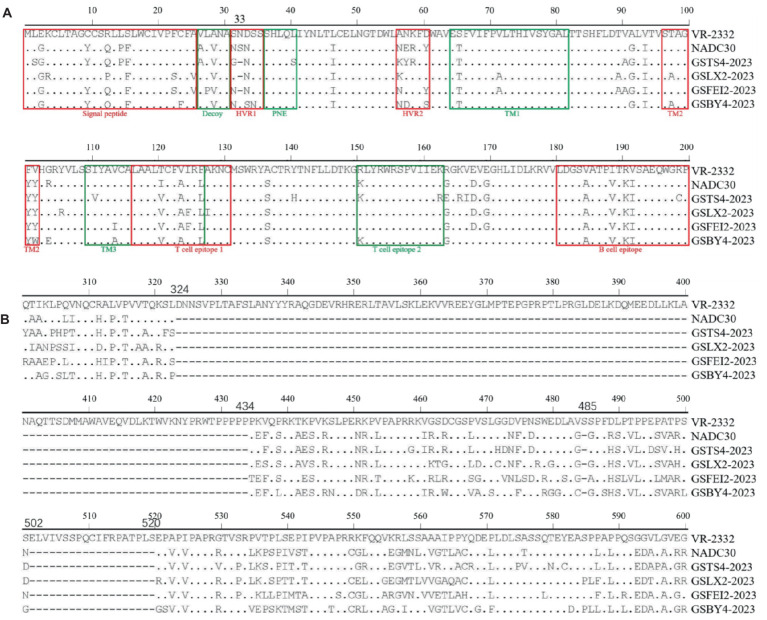
Alignment of the translated amino acid sequence among NADC30-like PRRSVs. **(A)** The GP5 protein. **(B)** The NSP2 protein.

Multiple amino acid substitutions were identified within various epitopes and hypervariable regions (HVR1 and HVR2) in ORF5 of the four strains. Several sites were substituted by the same amino acid among the four strains compared with VR-2332 (Table 2). Notably, one amino acid deletion at position 33 in HVR1 of ORF5 was found in GSTS4-2023, GSLX2-2023 and GSFEI2-2023, which was observed for the first time (Figure 2A). For NSP2, the four strains exhibited similar characteristics. Each of the four strains had a total length of 3,195 bases and encoded 1,064 amino acids. Amino acid sequence alignment revealed a discontinuous deletion of 131 amino acids (aa), also observed in NADC30, located at positions 322 ~ 432 aa, 483 aa and 493 ~ 511 aa with reference to VR-2332 (Figure 2B).

**Table 2:** The same amino acid substitutions of GP5 protein between the four strains obtained in Gansu and VR-2332.

Strains	Location													
	3	16	29	66	92	94	101	124	127	137	185	189	191	192
VR-2332	S	S	A	S	A	V	F	A	F	A	V	I	R	V
Gansu strains	G	F	V	T	G	I	Y	A	L	S	A	V	K	I

### Different recombination events revealed in the four strains

**Figure 3:**
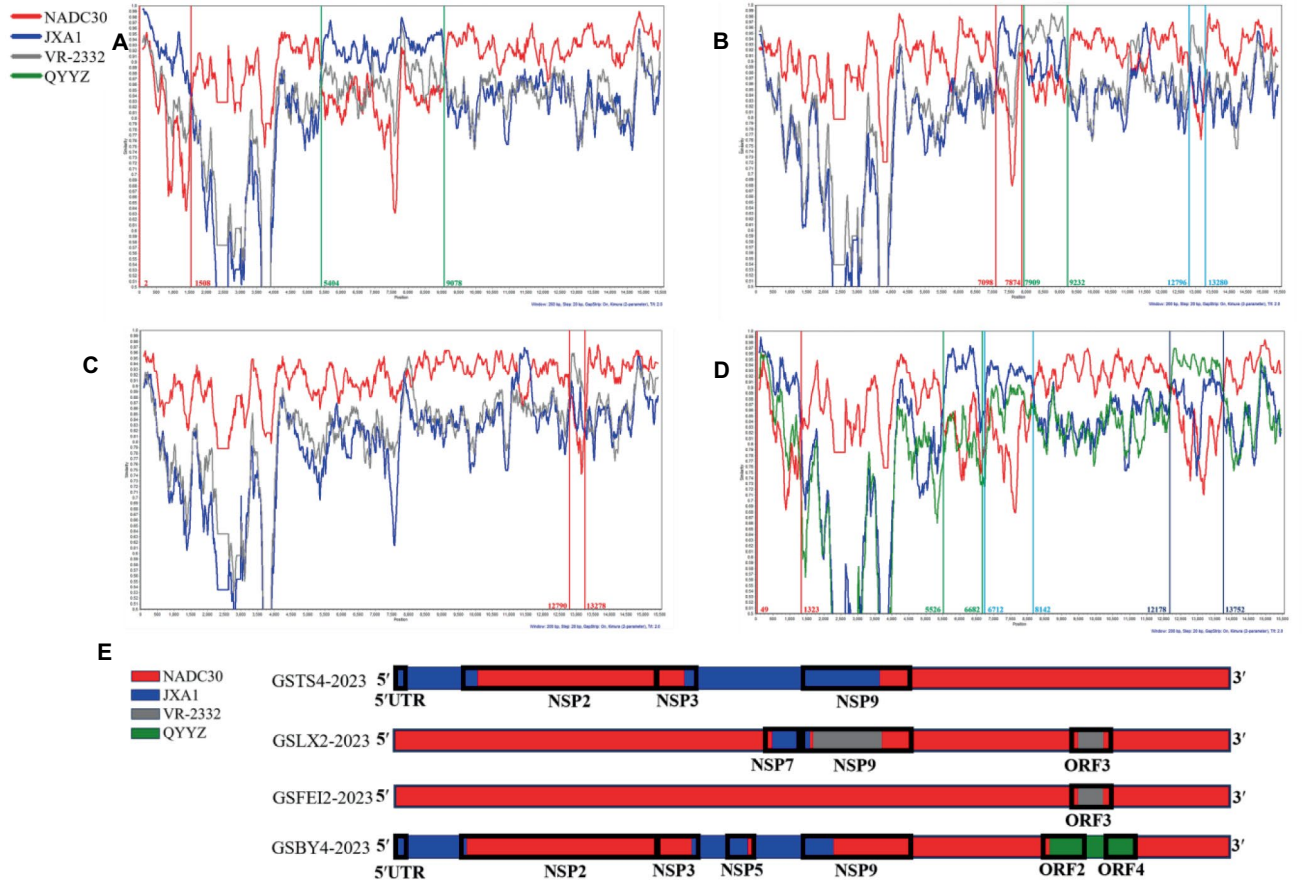
Recombination analysis of GSTS4-2023 **(A)**, GSLX2-2023 **(B)**, GSFEI2-2023 **(C)** and GSBY4-2023 **(D)**. The parent strains were NADC30 (red), JXA1 (blue), VR-2332 (gray) and QYYZ (green). Recombination events are marked within lines with identical color, and the recombination breakpoints are annotated with the same colors at the bottom with nucleotide positions consistent with VR-2332. **(E)** Simplified illustrations showing the location of the recombination breakpoints.

Recombination signal was detected for all four strains, at least 6 detection methods in RDP software showed a high degree of reliability in recombination, with *p*-values lower than 3.443 × 10^−8^, exhibiting a high extent of intra-lineage recombination. As expected, NADC30 was the major parent of the four strains, and the minor parents were JXA1-like strains, VR-2332-like strains and QYYZ-like strains (Table 3). Simplot analysis was performed to confirm the results from RDP4. Analyses results showed that four recombination breakpoints were detected in GSTS4-2023, which divided its whole genome into four segments related to NADC30-like strains and JXA1-like strains (Figure 3A). In addition, six breakpoints were revealed in GSLX2-2023, which is a recombinant product of JXA1-like strains and VR-2332-like strains (Figure 3B). In addition, two breakpoints were identified within GSFEI2-2023, which separated its whole genome into three parts related to NADC30-like strains and VR-2332-like strains (Figure 3C). Interestingly, six breakpoints were identified in GSBY4-2023, and the recombination events were between NADC30-like strains, JXA1-like strains and QYYZ-like strains (Figure 3D). Collectively, all of the four strains are recombinant strains with different recombination patterns, which were displayed in Figure 3E.

**Table 3:** Information of recombination events of GSTS4-2023, GSLX2-2023, GSFEI2-2023 and GSBY4-2023 revealed by RDP4.

Recombinant	Major parent	Minor parent			*p*-value of the detection methods						
			Beginning	Ending	RDP	GENECONV	BootScan	MaxChi	Chimera	SiScan	3Seq
GSTS4-2023	NADC30	JXA1	2	1,508	1.175 × 10^−64^	1.591 × 10^−13^	9.980 × 10^−62^	8.553 × 10^−225^	1.339 × 10^−25^	4.493 × 10^−22^	1.183 × 10^−34^
	NADC30	JXA1	5,404	9,078	5.324 × 10^−70^	9.860 × 10^−10^	6.882 × 10^−46^	1.178 × 10^−230^	3.214 × 10^−38^	1.995 × 10^−27^	2.932 × 10^−71^
GSLX2-2023	NADC30	JXA1	7,098	7,874	1.656 × 10^−43^	1.266 × 10^−22^	5.106 × 10^−43^	1.833 × 10^−16^	1.454 × 10^−17^	3.443 × 10^−08^	3.430 × 10^−12^
	NADC30	VR-2332	7,909	9,232	6.143 × 10^−41^	6.097 × 10^−22^	5.655 × 10^−40^	2.872 × 10^−20^	8.640 × 10^−23^	2.147 × 10^−18^	5.329 × 10^−14^
	NADC30	VR-2332	12,796	13,280	3.882 × 10^−28^	2.584 × 10^−11^	4.310 × 10^−28^	7.471 × 10^−12^	6.036 × 10^−12^	6.327 × 10^−11^	2.970 × 10^−200^
GSFEI2-2023	NADC30	VR-2332	12,790	13,278	1.247 × 10^−29^	7.627 × 10^−10^	1.074 × 10^−29^	5.278 × 10^−11^	4.479 × 10^−12^	9.534 × 10^−10^	1.554 × 10^−14^
GSBY4-2023	NADC30	JXA1	49	1,323	1.120 × 10^−38^	NS	4.949 × 10^−37^	4.358 × 10^−19^	4.906 × 10^−21^	6.857 × 10^−17^	8.884 × 10^−11^
	NADC30	JXA1	5,526	6,682	3.000 × 10^−47^	4.814 × 10^−19^	4.038 × 10^−48^	1.634 × 10^−17^	6.570 × 10^−20^	5.095 × 10^−18^	1.830 × 10^−20^
	NADC30	JXA1	6,712	8,142	9.943 × 10^−32^	NS	6.070 × 10^−30^	1.380 × 10^−29^	1.955 × 10^−31^	6.202 × 10^−19^	6.478 × 10^−51^
	NADC30	QYYZ	12,178	13,752	4.328 × 10^−56^	3.174 × 10^−32^	4.899 × 10^−57^	2.270 × 10^−25^	6.919 × 10^−25^	1.183 × 10^−25^	4.385 × 10^−18^

### GSTS4-2023, GSLX2-2023, and GSFEI2-2023 manifested low-level replication in cell culture

**Figure 4:**
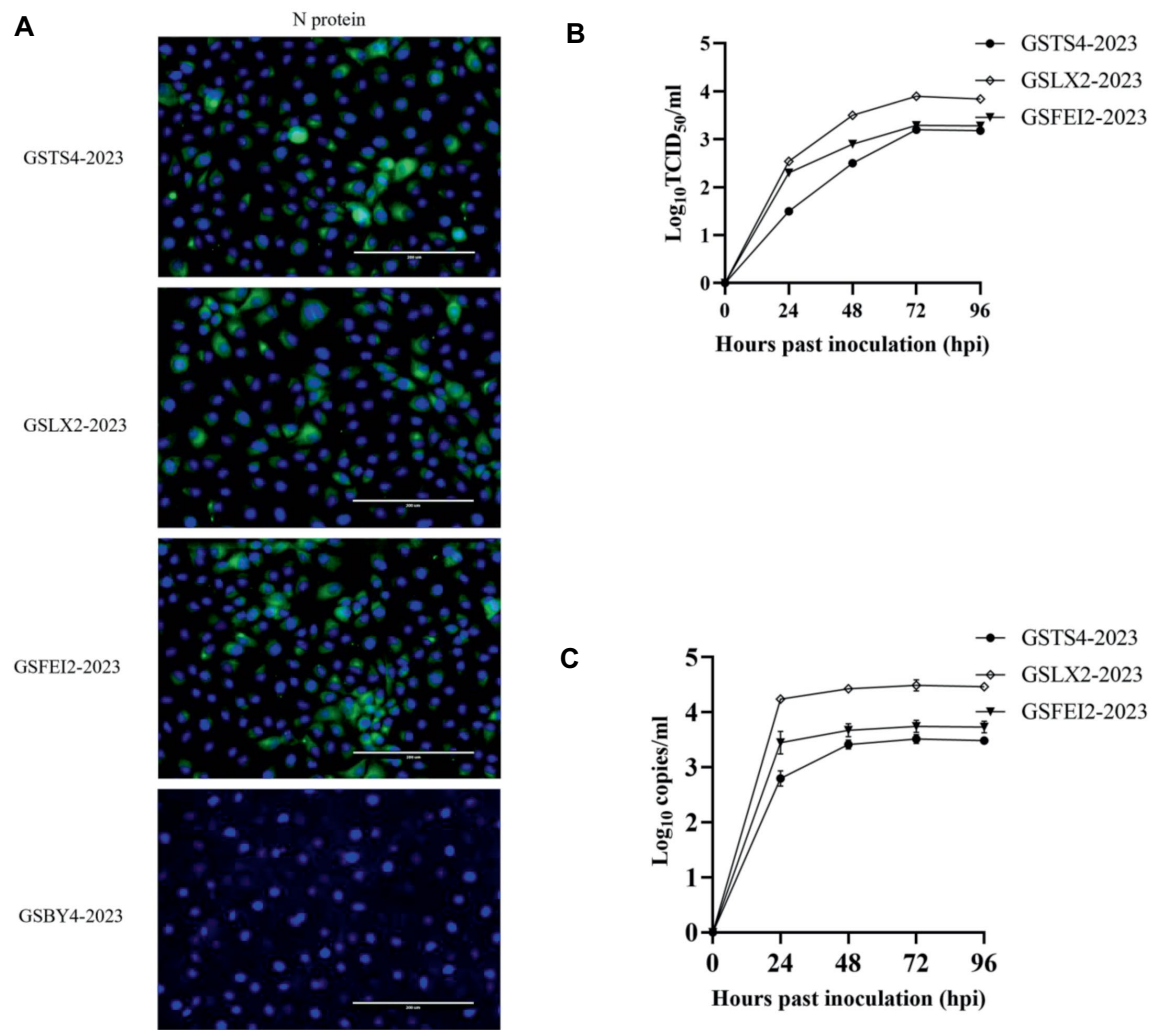
**(A)** IFA analysis of GSTS4-2023, GSLX2-2023, GSFEI2-2023 and GSBY4-2023 on MARC-145 cells with monoclonal antibodies against PRRSV N protein. **(B)** Virus titers of GSTS4-2023, GSLX2-2023 and GSFEI2-2023 at 24, 48, 72 and 96 hpi. **(C)** Virus load of GSTS4-2023, GSLX2-2023 and GSFEI2-2023 at 24, 48, 72 and 96 hpi.

Virus isolation was performed to further study the biological characteristics of the four strains. IFA revealed that Marc-145 cells inoculated with GSTS4-2023, GSLX2-2023, and GSFEI2-2023 produced a distinct green fluorescent signal, indicating their successful isolation (Figure 4A). The three isolates showed a relatively low replication ability according to TCID_50_ assay, especially for GSTS4-2023 (Figure 4B). Additionally, the copy number of the three strains were displayed in Figure 4C.

## Discussion

The initial outbreak of PRRS was reported in 1995 in north China (19). In the past few decades, PRRS has always been a major threat to the swine industry. Currently, it has been widely accepted that vaccination is the most effective method against this infectious disease. However, the development of vaccines with broad protection against multiple variants remains a major challenge for PRRS prevention and control due to frequent mutations and recombination events in the genome of PRRSV (30–32). Currently, PRRSV-2 is more virulent and widely distributed in China (20, 24, 33).

Recombination is a pervasive strategy for PRRSVs to maintain genetic diversity, especially in NADC30-like strains. Considering the high possibility of recombination in NADC30-like PRRSVs, numerous recombination events of the four NADC30-like strains were detected and confirmed by RDP4 and SimPlot in the present study. The numbers of recombination events detected in GSTS4-2023, GSLX2-2023, GSFEI2-2023 and GSBY4-2023 were 2, 3, 1 and 4, respectively. To date, recombination events between lineage 1 (as major parent) and other lineages (as minor parents) have been documented in several articles (24, 34, 35). The whole genome tree phylogenetically classified the four strains as NADC30-like strains into different minor branches, while there were multiple hallmarks of inter-lineage recombination in these strains. Short fragments of strains from other lineages recombine with NADC30, therefore the recombinant strains are scatter across different branches within the NADC30 clade. In this study, NADC30 was the major parent of all the four strains, and the minor parents were JXA1, VR-2332 and QYYZ, which belong to lineage 8, lineage 5 and lineage 3, respectively. The first lineage 8 strain, HP-PRRSV, was described in 2007 and spread rapidly throughout China. The emergence of lineage 5 strains was possibly caused by the extensively used commercial modified live vaccine (MLV) based on VR-2332. Currently, to our knowledge, lineage 3 strains (represented by QYYZ) have never been reported in Gansu province, which is located in northwest China.

QYYZ-like strains were first identified in Taiwan and Hong Kong, and spread to Guangdong in 2010 (10, 36). So far, QYYZ-like strains were detected subsequently in Guangdong, Fujian, Guangxi, Jiangxi, Shanghai, Xinjiang, Zhejiang, Heilongjiang, Sichuan, Guizhou and Henan (20, 24, 37, 38). Although we did not identify QYYZ-like strains in Gansu in the present study, a recombinant strain, GSBY4-2023, of NADC30 and QYYZ was characterized. This provided initial evidence for the potential existence of lineage 3 strains in Gansu province in the northwest of China.

PRRSVs have been phylogenetically divided into different lineages based on ORF5, which is related to the recognition of cellular receptor and virus neutralization (39). In this paper, the four strains were classified as NADC30-like strains belonging to lineage 1 based on ORF5. In addition, GSLX2-2023 and GSFEI2-2023 were classified into one individual minor branch, which is a clear evidence of virus evolution and continuous genomic changes of PRRSVs. Moreover, the absence of one N-linked glycosylation site (NGS) was detected in ORF5, and located in HVR1. The change of NGSs, which is known to associate with virus invasion and release, may enhance virulence and adaptability of PRRSVs (40, 41). Currently, amino acid changes introduced by substitution or recombination are common in HVR1, few articles reported amino acid deletion occurred in GP5 protein. In this research, one shared amino acid deletion in GP5 was observed in three NADC30-like PRRSV strains, which may become a new feature of NADC30-like PRRSV evolution, and further studies are needed to investigate the effects of this change on virus pathogenicity and replication.

NSP2 is another important viral protein with highly variable regions. Compared with VR-2332, multiple patterns of amino acid deletion were found, which can be used as a marker to distinguish between different PRRSV types (33). In the phylogenetic analysis of NSP2, the four strains were classified as NADC30-like strains, and shared the highest nucleotide homology with NADC30. The four strains were identified with a discontinuous 131-aa deletion (111aa + 1aa + 19aa) in NSP2, which is consistent with NADC30. As for the function of the aa deletions in NSP2, previous articles reported that the aa deletion could lead to a compact protein conformation of NSP2, which could help PRRSVs to evade host immune responses (42, 43). In fact, among wild-type PRRSVs within different lineages, NADC30-like strains have the highest number of amino acid deletions in NSP2 (typical 131-aa deletion), which may contribute to the widespread of NADC30-like strains (44).

ORF3 encodes GP3, a minor glycosylated structural protein that is essential for PRRSV infectivity and may be related to viral neutralization (45). Interestingly, since 2014, new hot spots for interlineage recombination occurred and were located in ORF3, which may be associated with the increased replication capacity and cell tropism to facilitate PRRSVs survival and spread (44). In our study, 3 of the 4 strains of lineage 1 had recombined with strains of other lineages in and around ORF3, indicating that the probability of recombination of ORF3 may increase and further complicate the evolution of PRRSVs.

The virulence of PRRSVs varies greatly and is mainly derived from its genetic characteristics. Strains from lineage 8 are more virulent than other lineages on average (15, 46). However, for NADC30-like strains, continuous mutation and extensive interlineage recombination may contribute to the variation of virulence. Particularly, R^13^ and R^151^ in the ORF5 protein have been reported to be two crucial sites contributing to the virulence of PRRSVs (47, 48). In the present study, R^13^ → Q^13^ or P^13^ occurred in three isolates, and R^151^ → K^151^ occurred in GSTS4-2023, which may lead to potential virulence changes in the isolates.

## Conclusion

In summary, four whole genomes of new NADC30-like PRRSV strains were described in detail, and they exhibited different genomic characteristics and recombinant patterns. One NGSs deletion in ORF5 was found in 3 of the 4 strains, which probably become a new trend of PRRSVs genome evolution. All of the four strains had the typical 131 amino acid deletion of NADC30-like PRRSV. The described genomic characteristics of the new NADC30-like strains further enriched the epidemiological information of PRRSV, and provided useful references for disease prevention and control.

## Data availability statement

The original contributions presented in the study are publicly available. This data can be found at: https://www.ncbi.nlm.nih.gov/; PP409066-PP409069.

## Ethics statement

The animal studies were approved by Animal Ethics Committee of Lanzhou Veterinary Research Institute of Chinese Academy of Agricultural Sciences (Permit No. LVRIAEC2016-008). The studies were conducted in accordance with the local legislation and institutional requirements. Written informed consent was obtained from the owners for the participation of their animals in this study.

## Author contributions

SJ: Writing – original draft, Writing – review & editing, Methodology, Software. JZ: Writing – review & editing, Data curation. JW: Writing – review & editing, Investigation. XM: Writing – review & editing, Methodology. GL: Writing – review & editing, Project administration. JL: Writing – review & editing, Formal analysis. ZC: Writing – review & editing, Validation. DL: Writing – review & editing, Visualization. PL: Writing – review & editing, Data curation. QZ: Writing – review & editing, Investigation. ZLi: Writing – review & editing, Data curation, Methodology. ZLu: Writing – review & editing, Methodology, Supervision. PS: Writing – review & editing, Investigation, Methodology, Supervision.

## Footnotes

^1^ https://services.healthtech.dtu.dk/services/NetNGlyc-1.0/

^2^ https://www.chiplot.online/
