# Supplementary material for: Whole-genome analysis of the recombination and evolution of newly identified NADC30-like porcine reproductive and respiratory syndrome virus strains circulated in Gansu province of China in 2023
Source: Front Vet Sci. 2024 Apr 12;11:1372032. doi: 10.3389/fvets.2024.1372032 (PMC11047440; doi:10.3389/fvets.2024.1372032)
Supplement: Supplementary file 1 [file Table_1.DOCX]

Supplementary table 1. Primers used for detection of PRRSVs.

| Name of sequence | Sequence of RT-PCR primers | Position in genome | Length of RT-PCR products |
| --- | --- | --- | --- |
| ORF5-F | GTGTCAGGCATYGTGGCTGTGTG | 13200 | 1091 |
| ORF5-R | CCTAGCAAGCACAAMCGGCATCTGG | 14290 |  |
