# Supplementary material for: Whole-genome analysis of the recombination and evolution of newly identified NADC30-like porcine reproductive and respiratory syndrome virus strains circulated in Gansu province of China in 2023
Source: Front Vet Sci. 2024 Apr 12;11:1372032. doi: 10.3389/fvets.2024.1372032 (PMC11047440; doi:10.3389/fvets.2024.1372032)
Supplement: Supplementary file 2 [file Table_2.DOCX]

Supplementary table 2. Primers used for amplification of the whole-genome of PRRSVs.

| Fragment | Sequence of RT-PCR primers | Position in genome | Length of RT-PCR products |
| --- | --- | --- | --- |
| A | ATGACGTATAGGTGTTGGCTC | 1 | 1330 |
|  | TCTCTTTCCBGCACCGTACCA | 1330 |  |
| B | GGCAAGTACCTNCAGCGGAGGCT | 1098 | 2713 |
|  | AAGGCTGCGYACAGGGTCCCAAGG | 3810 |  |
| C | GGTGTATTTTCTGGGTCTTCTCGG | 3647 | 2018 |
|  | TACAAAACTGGCCTGAGGGG | 5664 |  |
| D | GGACTTCGCCATAGCYGATTGCC | 5413 | 2214 |
|  | ATCCCAAAGCGTGCCATCAATCCC | 7626 |  |
| E | GGCGGCTTRGTTGTTACTGAGAC | 7356 | 1961 |
|  | GGACAATGCTGGTGRAAGTG | 9316 |  |
| F | CCATGTGGGAAAAACTCAGGTC | 9206 | 1951 |
|  | AGTCCAACACADTTTCCAGCAC | 11156 |  |
| G | CTTCCGCGCTTCCTTCCCAAG | 11000 | 2396 |
|  | CGCGGTCAAGCAYTTCCCCAACATA | 13395 |  |
| H | GTGTCAGGCATTGTGGCTGTGTG | 13200 | 1801 |
|  | TTTTTTTTTTTTTTTTTTTTTAATTDCGGCCGCATGGTTCTCG | 15000 |  |
