# Supplementary material for: Whole-genome analysis of the recombination and evolution of newly identified NADC30-like porcine reproductive and respiratory syndrome virus strains circulated in Gansu province of China in 2023
Source: Front Vet Sci. 2024 Apr 12;11:1372032. doi: 10.3389/fvets.2024.1372032 (PMC11047440; doi:10.3389/fvets.2024.1372032)
Supplement: Supplementary file 3 [file Table_3.DOCX]

Supplementary table 3. Primers used for amplification of the 5' UTR and 3' UTR of PRRSVs.

| Fragment | Name of sequence | Sequence of RT-PCR primers |
| --- | --- | --- |
| I | 5'-GSP1 | TAGGGACAGTACCAGCGGGGAGGCAGCC |
|  | 5'-NGSP1 | GGGGAGGCAGCCGTTTTGGTAC |
| J | 5'-GSP2 | TTTGGGCCTCTGCGGGAGCGGCAAGTTG |
|  | 5'-NGSP2 | GAGCGGCAAGTTGGTCAACACAT |
| K | 3'-GSP1 | ATGACAGCACAGCCCCGCAAAAGGTGCT |
|  | 3'-NGSP1 | AAGGTGCTTTTGGCGTTTTCC |
| L | 3'-GSP2 | GGGGAGTGGCCAGCCAGTCAATCAGCTG |
|  | 3'-NGSP2 | GTCAATCAGCTGTGCCAGATGT |
