# Supplementary material for: Whole-genome analysis of the recombination and evolution of newly identified NADC30-like porcine reproductive and respiratory syndrome virus strains circulated in Gansu province of China in 2023
Source: Front Vet Sci. 2024 Apr 12;11:1372032. doi: 10.3389/fvets.2024.1372032 (PMC11047440; doi:10.3389/fvets.2024.1372032)
Supplement: Supplementary file 4 [file Table_4.DOCX]

Supplementary table 4. Reference PRRSV strains used in this study.

| No. | Name | Country | Year | Accession number |
| --- | --- | --- | --- | --- |
| 1 | HUN4 | China | 2007 | EF635006 |
| 2 | JXA1 | China | 2006 | EF112445 |
| 3 | HB-1(sh)/2002 | China | 2002 | AY150312 |
| 4 | HB-2(sh)/2002 | China | 2004 | AY262352 |
| 5 | BJ-4 | China | 2000 | AF331831 |
| 6 | VR-2332 | USA | 1992 | AY150564 |
| 7 | NADC30 | USA | 2008 | JN654459 |
| 8 | GS2002 | China | 2002 | EU880441 |
| 9 | QYYZ | China | 2010 | JQ308798 |
| 10 | HENZMD-9 | China | 2015 | KU950374 |
| 11 | CH-1R | China | 2008 | EU807840 |
| 12 | FJFS | China | 2012 | KP998476 |
| 13 | TJnh1501 | China | 2015 | KX510269 |
| 14 | LNWK130 | China | 2017 | MG913987 |
| 15 | CH/2018/NCVAnheal-1 | China | 2018 | MH370474 |
| 16 | FJ0908 | China | 2018 | MK202794 |
| 17 | HLJZD22-1812 | China | 2018 | MN648450 |
| 18 | HLJDZD32-1901 | China | 2019 | MN648449 |
| 19 | CH/SCCD-4/2020 | China | 2020 | OL771206 |
| 20 | CH/SCLS-2/2020 | China | 2020 | OL771207 |
| 21 | CH/SCMS-4/2020 | China | 2020 | OL771208 |
| 22 | CH/SCMY-2/2019 | China | 2019 | OL771205 |
| 23 | BJ0706 | China | 2007 | GQ351601 |
| 24 | CH-1a | China | 1996 | AY032626 |
| 25 | Gansu-2017-51 | China | 2019 | MN046235 |
| 26 | GS2004 | China | 2009 | EU880443 |
| 27 | GSWW/2018 | China | 2018 | OP764591 |
| 28 | GSWW/CHA/2015 | China | 2015 | KX767091 |
| 29 | GZgy15-1 | China | 2015 | KT358728 |
| 30 | HBFL-1604 | China | 2018 | MH651739 |
| 31 | HPBEDV | China | 2007 | EU236259 |
| 32 | IA/2014/NADC34 | USA | 2014 | MF326985 |
| 33 | IA/2015/NADC35 | USA | 2015 | MF326986 |
| 34 | ISU30 | USA | 2014 | KT257977 |
| 35 | SHB | China | 2005 | EU864232 |
| 36 | XL2008 | China | 2008 | EU880436 |
| 37 | MN184A | USA | 2005 | DQ176019 |
| 38 | MN184C | USA | 2007 | EF488739 |
