# Supplementary material for: Whole-genome analysis of the recombination and evolution of newly identified NADC30-like porcine reproductive and respiratory syndrome virus strains circulated in Gansu province of China in 2023
Source: Front Vet Sci. 2024 Apr 12;11:1372032. doi: 10.3389/fvets.2024.1372032 (PMC11047440; doi:10.3389/fvets.2024.1372032)
Supplement: Supplementary file 5 [file Table_5.DOCX]

Supplementary table 5. Primers and Taqman probe used for RT-qPCR.

| Name of primers/probes | Sequence of RT-qPCR primers/probes |
| --- | --- |
| PRRSV-rF14659 | CGGCAARTGATAACCACGC |
| PRRSV-rR14743 | TTCTGCCACCCAACACGAG |
| PRRSV-rProbe | FAM-TGTGCCGTTRACCGTAGTRGAGCC-TAMRA |
